# Cytomegalovirus and Epstein-Barr Virus in Breast Cancer

**DOI:** 10.1371/journal.pone.0118989

**Published:** 2015-02-27

**Authors:** Ann K. Richardson, Margaret J. Currie, Bridget A. Robinson, Helen Morrin, Yen Phung, John F. Pearson, Trevor P. Anderson, John D. Potter, Logan C. Walker

**Affiliations:** 1 Wayne Francis Cancer Epidemiology Research Group, School of Health Sciences, University of Canterbury, Christchurch, New Zealand; 2 Mackenzie Cancer Research Group, University of Otago, Christchurch, New Zealand; 3 Biostatistics and Computational Biology Unit, University of Otago, Christchurch, New Zealand; 4 Canterbury Health Laboratories, Christchurch, New Zealand; 5 Public Health Sciences Division, Fred Hutchinson Cancer Research Center, Seattle, United States of America; 6 Centre for Public Health Research, Massey University, Wellington, New Zealand; University of North Carolina at Greensboro, UNITED STATES

## Abstract

Findings of polymerase chain reaction (PCR) studies of cytomegalovirus (CMV) and Epstein-Barr virus (EBV) and breast cancer vary, making it difficult to determine whether either, both, or neither virus is causally associated with breast cancer. We investigated CMV and EBV in paired samples of breast cancer and normal breast tissue from 70 women using quantitative PCR. A serum sample from each woman was tested for CMV and EBV IgG. To place our results in context, we reviewed the existing literature and performed a meta-analysis of our results together with previous PCR studies of EBV, CMV, and breast cancer. Of the serology samples, 67 of 70 (96%) were EBV IgG positive and 49 of 70 (70%) were CMV IgG positive. QPCR detected EBV in 24 (34%) of the tumour and 9 (13%) of the paired normal specimens and CMV in 0 (0%) of the tumour and 2 (3%) of the paired normal specimens. Our findings, together with earlier results summarised in the meta-analysis, suggest several possibilities: variable findings may be due to limitations of molecular analyses; ‘hit and run’ oncogenesis may lead to inconsistent results; one or both viruses has a role at a later stage in breast cancer development; infection with multiple viruses increases breast cancer risk; or neither virus has a role. Future studies should focus on ways to investigate these possibilities, and should include comparisons of breast cancer tissue samples with appropriate normal tissue samples.

## Introduction

Breast cancer is the most commonly diagnosed cancer in women worldwide.[1] Several human cancers can be caused by viruses and a virus, mouse mammary tumour virus (MMTV), causes breast cancer in mice.[2] We hypothesised that late exposure (in adulthood rather than in childhood) to a common virus such as CMV or EBV may cause breast cancer.[3, 4] Cytomegalovirus is ubiquitous in human populations but patterns of exposure differ among countries. Breast cancer incidence is low in those countries where most people seroconvert in childhood and where therefore nearly 100% of adults are CMV-seropositive. Breast cancer incidence is highest in countries where exposure to CMV may occur late, with a strong inverse correlation (Pearson correlation coefficient −0.79, p < 0.0001) between breast cancer incidence and the proportion of CMV-seropositive adults in various countries.[3]

In a case-control study of CMV and EBV and breast cancer,[5] mean CMV IgG levels were higher in cases than controls, with an adjusted odds ratio (OR) per unit increase in CMV IgG of 1.46 and 95% confidence interval (CI) 1.06–2.03. To investigate whether CMV or EBV IgG levels were elevated before the diagnosis of breast cancer, a nested case-control study with two serum samples taken at least four years before the diagnosis of breast cancer in cases, and samples matched for duration of storage from controls, were tested for CMV and EBV IgG.[6] The risk of breast cancer, adjusted for parity, was greater per unit difference in CMV IgG between samples: OR 1.7 (95% CI 1.1–2.5). In an analysis restricted to parous cases and age-matched parous controls, the OR for seroconversion between samples in the same individual, adjusted for parity and age at first birth, was 9.7 (95% CI 1.2–77.3). Both case-control studies found that higher IgG levels (possibly indicating late exposure to CMV) are associated with breast cancer but there was no association between EBV IgG levels and breast cancer.

CMV may have a role as an ‘oncomodulator’ in changing the tumour microenvironment as well as in initiation and promotion of tumour cells.[7–10] PCR has been used to investigate CMV in breast tumour and normal tissue,[11–13] with CMV genetic material found in a higher proportion of tumour tissue than normal tissue. The role of EBV in breast cancer is controversial. [14–19] A case-control study linked delayed EBV infection with breast cancer,[4] and a recent study using QPCR suggested EBV may be a marker of biological aggressiveness in breast cancer.[20] Three earlier studies also suggested that breast cancer may be associated with late infection and/or immune response to late infection.[21–23] There is evidence for both CMV and EBV as disruptors of telomere maintenance, which may play a role in cancer development. [24, 25] However, two earlier studies using QPCR of EBV and breast cancer found very low levels of EBV DNA in breast cancer tissue[26, 27] and a recent study using immunohistochemistry (IHC) and in situ hybridization (ISH) found EBV in infiltrating lymphocytes in breast cancer tissue but not in malignant cells.[28]

Using quantitative PCR (QPCR), we compared CMV and EBV genetic material in paired samples of breast cancer and histologically normal breast tissue from 70 women. We also measured serum CMV and EBV IgG levels. In addition, we reviewed the existing literature on PCR studies of EBV and/or CMV and breast cancer and performed a meta-analysis of our results together with earlier findings for EBV, CMV, and breast cancer.

## Materials and Methods

Tissue and serum samples collected and stored by the Cancer Society Tissue Bank, Christchurch (CSTB) were analysed. All patients attending Christchurch Hospital, New Zealand, for cancer-related surgery are potential CSTB donors. Written informed consent (Ethics Committee Approvals 02.06.98/5.11.09) was obtained for the collection, storage, and use of all samples. Standard CSTB procedures were followed; these include culturally appropriate tissue handling and disposal protocols. [29] Each sample in the CSTB has a computer-generated unique identifier to protect patient confidentiality and is accompanied by extensive clinicopathological data and recent follow-up data. Approval for this project was granted by the University of Otago Human Ethics Committee (UOHEC 13/079) and the Canterbury Tissue Bank Board (13.03.2013).

Serum was prepared from blood drawn by venepuncture at least 24 hours prior to surgery and stored at -80°C. Breast tissue from surgery was evaluated by an experienced histopathologist, who selected representative tumour and histologically normal samples for storage. Normal tissue samples were dissected from areas that showed no fibrosis and were at least 70–100 mm away from the tumour. Fresh tissues were placed in a storage cryovial, snap frozen in liquid nitrogen, and stored at -80°C in <50 minutes from surgical removal.

Sample-size calculations showed that 60 paired samples would allow us, using QPCR, to detect a difference between breast cancer and normal breast DNA in mean copies per ml of ± 0.37 standard deviations, with 95% confidence and 80% power. We prepared 70 paired samples to allow for failure of processing. The breast cancer samples included a range of grade, receptor status, and disease stage (Table 1 shows the characteristics of the patients and tissue samples).

**Table 1:** Patient and tumour characteristics.

Age of Patient at Surgery	Percent
25–29	1.3
30–34	2.6
35–39	5.2
40–44	20.8
45–49	7.8
50–54	13.0
55–59	3.9
60–64	9.1
65–69	6.5
70–74	7.8
75–79	6.5
80–84	6.5
85+	9.1
Tumour Grade	Percent
1	8.5
2	29.6
3	62.0
Histologic Type	Number
Infiltrating ductal carcinoma	70
Normal (paired samples from women with breast cancer)	70
Surgical Type	Percent
Mastectomy	8.1
Mastectomy and axillary dissection/clearance	82.5
Wide local excision and axillary dissection	9.5

### DNA isolation and quantitative PCR (QPCR)

DNA from invasive breast cancer tissue and from paired normal breast tissue was extracted from frozen samples. DNA was isolated using the QIAamp DNA mini kit (Qiagen) using the addition of polyA oligonucleotides (Roche Diagnostics) as described by the manufacturer. The manufacturer recommends that carrier DNA (for example poly dA) is used when the sample is low-copy (when <10,000 copies are present). This step was important for efficient recovery of viral genomes. Quantitation of CMV and EBV DNA was carried out using CMV- and EBV-specific primer sequences targeted to the conserved pp65[30] and EBNA-1 (Qiagen Artus EBV TM PCR kit) regions, respectively. The DNA sequence of primer pairs and Taqman probes designed for each target region are shown in Table 2. The CMV and EBV quantitation standards were created by a plasmid construct of the PCR product and CMV copy number was determined by droplet digital PCR (Bio-rad). We used reference human DNA sequence from the *ALB* gene—which is known to lack germline copy number variation—to validate DNA isolation and perform comparative analysis between normal and breast cancer tissue samples. All PCR reactions were performed on an ABI 7500 real-time PCR system (Applied Biosystems, Life technologies) and null PCR results were validated by an inhibitor control target included in the EBV kit.

**Table 2:** Sequences of primers and probes used for QPCR.

Target	Forward primer (5’-3’)	Reverse primer (5’-3’)	Taqman Probe (5’-3’)
**CMV—pp65**	GCAGCCACGGGATCGTACT	GGCTTTTACCTCACACGAGCATT	CGCGAGACCGTGGAACTGCG
**EBNA-1**	Unpublished*	Unpublished*	Unpublished*
***ALB***	GCTGTCATCTCTTGTGGGCTGT	AAACTCATGGGAGCTGCTGGTT	CCTGTCATGCCCACACAAATCTCTCC

* The Artus EBV TM PCR kit primer and probes are proprietary and not made publicly available.

### Serology

A stored serum sample from each woman was extracted to establish CMV and EBV IgG serostatus and compare these with the QPCR results. Serum samples were tested at Canterbury Health Laboratories for seropositivity to CMV and EBV using standard enzyme immunoassays (Euroimmun, Luebeck, Germany) for CMV IgG and EBV viral capsid antigen (VCA) IgG, with measurement in units of optical density.

### Statistical analysis

Random effects meta-analysis was performed on the proportions of samples positive for EBV and CMV respectively rather than relative risks, because only six studies (including ours) included paired normal samples. Proportions had 95% confidence intervals derived from the Normal approximation to the binomial with 0.5 added to zero counts. Estimates of average proportion positive were calculated for all studies and for each assay type that was used in multiple studies; however, there was considerable heterogeneity in both EBV and CMV analyses (I^2^ = 98.6% and 99.6% respectively). Analysis was performed in R 3.1.1 (Vienna, Austria) using the Metafor package.[31]

## Results and Discussion

The age range of the women studied was 25–88, with most in the 40–54 year age-group (Table 1). All had infiltrating ductal carcinoma, with 62% high-grade tumours. The vast majority of tissue samples (90.5%) were from women who had undergone mastectomy, because we needed invasive breast cancer tissue and normal breast tissue samples that were separated by at least 70mm. Most tissue samples from wide-local excisions were unsuitable for obtaining paired samples because there was not enough normal tissue available that was >70mm from the tumour.

QPCR detected EBV in 24 (34%) of the 70 tumour specimens and in 9 (13%) of the paired normal specimens. CMV was detected in 0 (0%) of the 70 tumour specimens and in 2 (3%) of the paired normal specimens (Table 3).

EBV positivity was not associated with grade, receptor status, or disease stage.

**Table 3:** Results of QPCR analysis.

	Breast cancer tissue		Paired normal tissue	
	Number	Percent	Number	Percent
CMV positive	0	0.0	2	2.9
CMV negative	70	100.0	68	97.1
EBV positive	24	34.3	9	12.9
EBV negative	46	65.7	61	87.1

Sixty-seven of 70 (96%) of the serology samples that corresponded to the paired tissue samples were EBV IgG positive. Forty-nine of 70 (70%) of the serology samples that corresponded to the paired tissue samples were CMV IgG positive. In general, CMV seropositive women were younger (mean age 51) than CMV seronegative women (mean age 62) p <0.01.

**Fig 1:**
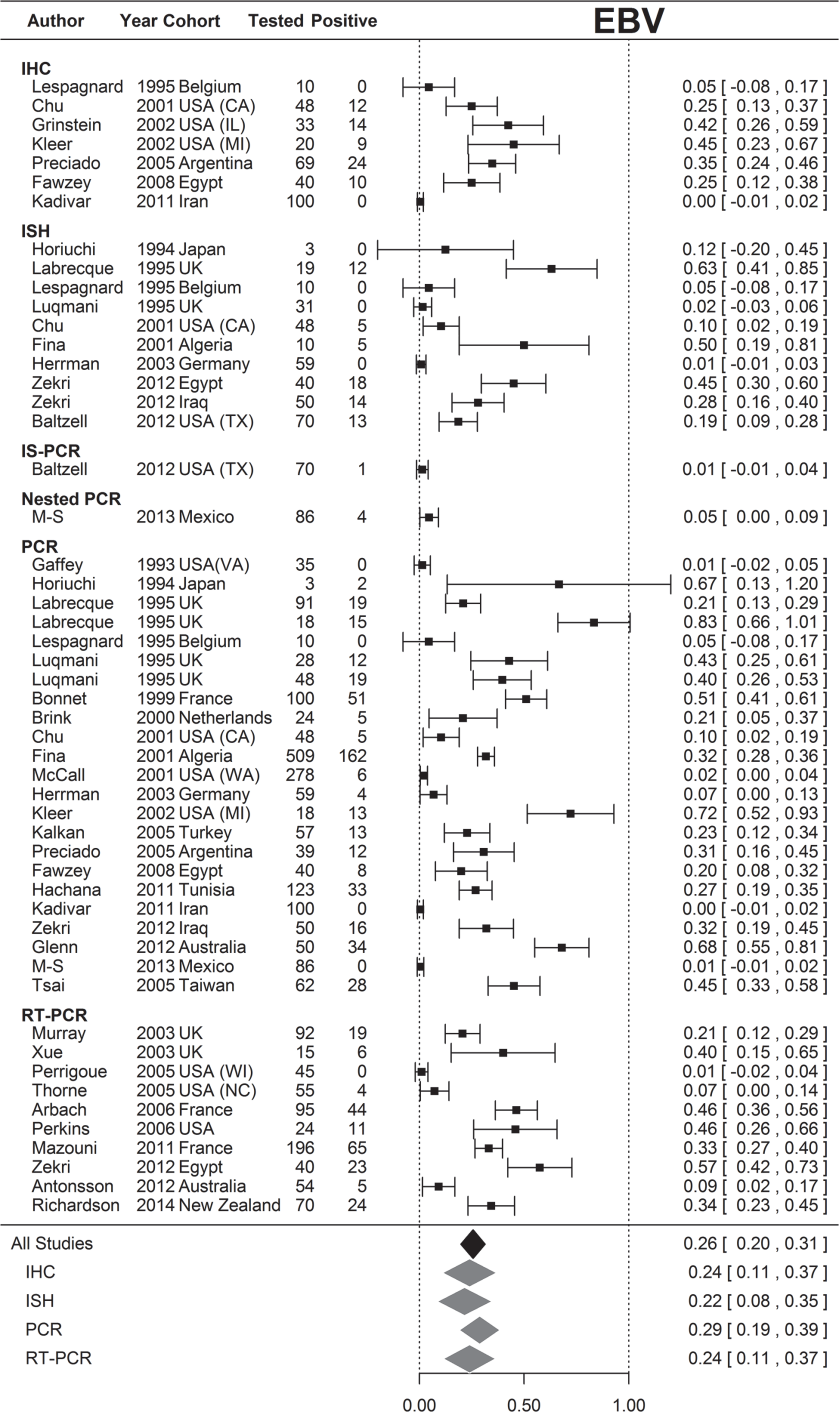
Meta-analysis of EBV positivity in breast cancer tissue samples. Random effects meta-analysis was performed on the proportions of samples that were positive for EBV rather than relative risks, because only six studies (including ours) included paired normal samples. Proportions had 95% confidence intervals derived from the Normal approximation to the binomial with 0.5 added to zero counts. Estimates of the average proportion positive were calculated for all studies and for each assay type that was used in multiple studies. Results of studies that analyzed breast tissue samples for EBV are shown according to the method of analysis. The overall strength of association for each type of analysis, and the overall strength of association for all studies are shown at the bottom of the figure. There was considerable heterogeneity in the EBV analyses (I^2^ = 98.6%).

**Fig 2:**
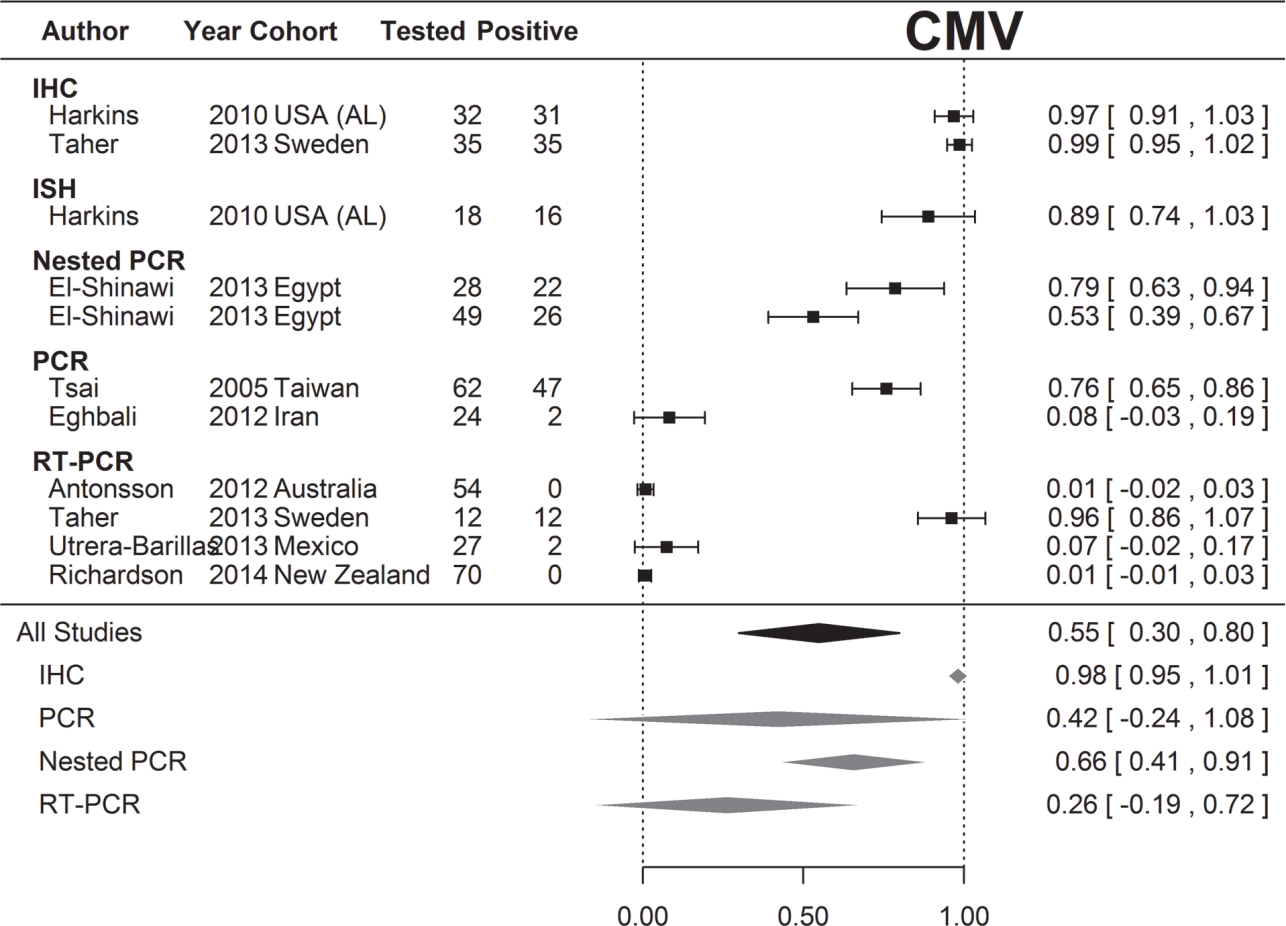
Meta-analysis of CMV positivity in breast cancer tissue samples. Random effects meta-analysis was performed on the proportions of samples that were positive for CMV. Proportions had 95% confidence intervals derived from the Normal approximation to the binomial with 0.5 added to zero counts. Estimates of the average proportion positive were calculated for all studies and for each assay type that was used in multiple studies. Results of studies that analyzed breast tissue samples for CMV are shown according to the method of analysis. The overall strength of association for each type of analysis, and the overall strength of association for all studies are shown at the bottom of the figure. There was considerable heterogeneity in the CMV analyses (I^2^ = 99.6%). Footnote to Fig. 2: PCR results for Harkins et al were not included in this meta-analysis because nested PCR was performed on only 8 specimens, all of which were positive on IHC (please see S1 Table).

Meta-analysis revealed considerable heterogeneity in positivity for both EBV (Fig. 1) and CMV (Fig. 2) in breast cancer populations. The average positivity for CMV sits between two modes. Our present study and three previously reported PCR- or QPCR-based studies produced positive proportions statistically indistinguishable from zero, whereas two found positivity to be very high or statistically indistinguishable from complete positivity (Fig. 2). All studies using IHC or ISH found CMV to be present in almost all participants. The mean positivity for EBV is 26%, (95% CI 20%-31%) with 12 out of 54 (22.2%) studies across all assay types detecting positivity statistically indistinguishable from zero whereas only two (3.7%) studies are consistent with complete positivity.

Published results of PCR analyses of CMV and/or EBV in breast cancer have been inconsistent. In this study, we performed an inhibitor control reaction to validate the null samples and used a house-keeping gene (ALB)-PCR to ensure DNA quality (amplifiable DNA present). All samples were positive for ALB DNA amplification, indicating that the results are interpretable as they stand. We did not use microdissection (which can cause virus levels to be too low to amplify) and used histologically normal cells with no visible signs of atypia, which meant that our study avoided some of the limitations of earlier studies. Only four previous studies have used paired breast cancer and normal breast tissue samples for PCR analysis of EBV[27, 32, 33] or CMV(13) and our study and one other[34] have included PCR analysis of both viruses in paired breast cancer and normal breast samples. To place our results in context, we undertook a comprehensive review of the existing literature on PCR studies of EBV and/or CMV and breast cancer, together with a meta-analysis. The findings are summarised in S1 Table and in Figs. 1 and 2 and discussed below.

Of 32 PCR studies of EBV and breast cancer, 22 (69%) had positive results ranging from 2% to 100% of specimens tested, [11, 14, 16, 18, 20, 26, 32, 35–49] whereas 10 studies (31%) did not[27, 33, 34, 50–56]. There are fewer PCR studies of CMV and breast cancer. Of seven studies, six (86%) found positive results, ranging from 7.4% to 100% of specimens tested, [11, 13, 57–60] whereas one study did not detect CMV in any of 54 samples but found EBV in 10% of the samples[34]. The results from the two studies by Tsai *et al* have been included once, as these studies used the same tissue samples.[11, 12] The extreme heterogeneity in the meta-analysis of CMV and EBV breast cancer studies is of great concern. Firstly, the extreme variation in positivity invalidates any inferences using mean positivity across breast cancer populations, particularly for CMV. Secondly, the spread of results between and within assay types, albeit on different populations, admits the possibility that the assays are not reliable. Again, this is particularly acute for CMV.

The primers used for PCR analysis have differed among studies but this does not explain the findings for EBV and breast cancer, as some studies using the same primer found positive results whereas others found null results. Two studies[27, 42] warned about false-positive results, noting that the PCR result for one lytically infected cell containing several hundred copies of the EBV genome would be indistinguishable from several hundred cells each containing a single copy of the genome[42] and that PCR techniques using many cycles of amplification could detect a single molecule of EBV DNA.[27] There have been fewer PCR studies of CMV and breast cancer but all that used IE-gene primers have been positive. IE-genes are important in allowing CMV to escape immunosurveillance and IE proteins have been shown to introduce mutations in cellular DNA, [61] whereas pp65 is not required for primary and persistent infection in animals.[62]

In situ hybridization (ISH) is regarded as superior to PCR because it can differentiate between viral infections in tumour cells and infection in other cells but its sensitivity and specificity depend on the target used. Positive findings for EBV were less likely with ISH than PCR: of 16 studies of EBV and breast cancer using both ISH and PCR, five (31%) found positive results with both PCR and ISH,[16, 36, 38, 39, 48] whereas nine (56%) found positive PCR but null ISH results,[14, 32, 35, 37, 45, 52–54, 56] and two (13%) found null results for both.[27, 51] One study of CMV and breast cancer, using PCR and ISH, found positive results for both.[13] Advantages and limitations of analyses for viruses in breast cancer are summarised in Table 4.

**Table 4:** Advantages and limitations of molecular analyses.

	Advantages	Limitations
Immunohistochemistry (IHC)	Can distinguish virus in tumour cells from virus in other cells such as lymphocytes.	May lack specificity due to cross-reactivity with cellular proteins (especially EBV EBNA1).
In-situ hybridization (ISH)	Can distinguish virus in tumour cells from virus in other cells such as lymphocytes.	The sensitivity and specificity of ISH depend on the target (high sensitivity and specificity for EBV EBERs).
Southern Blot hybridization	Permits semi-quantification of viral load.	Less sensitive than PCR for detecting viral DNA. Partial deletion or polymorphism of the viral DNA could prevent hybridization to viral DNA.
Polymerase chain reaction (PCR)	Highly sensitive and specific method for detecting the presence of viral DNA.	Cannot differentiate between cell types (for instance in breast tumours with lymphocytic infiltrates). Use of laser capture microdissection (LCM) may cause virus levels to be too low to amplify using PCR. Possibility of false positive results due to lytic viral replication.
Nested PCR	Highly sensitive and specific method for detecting the presence of viral DNA, with enhanced amplification.	Prone to contamination (for example by positive control DNA).
Quantitative polymerase chain reaction (QPCR)	Highly sensitive and specific method for detecting the presence of viral DNA, which allows quantification of viral DNA.	LCM can cause virus levels to be too low to quantify using QPCR. QPCR may be prone to contamination, but this is less likely than with standard PCR.

[46, 63, 64].

The type of specimen tested does not explain differing results for EBV: although 12 (71%) studies found positive PCR results in formalin-fixed, paraffin-embedded specimens, [17, 35, 37, 38, 40, 43, 45, 47, 48, 54, 56] six did not. [27, 43, 51, 55, 65] One study, using both frozen and formalin-fixed, paraffin-embedded specimens, was negative with PCR but 5% of specimens were positive with nested PCR.[33] Four studies of CMV using formalin-fixed, paraffin-embedded specimens found positive results, ranging from 7.4% to 100% of specimens tested,[13, 57, 59, 60] whereas one study, using frozen samples was positive [11] and our study and one other, [34] using frozen samples, had null results. One study using fresh breast cancer tissue samples was positive.[58]

Similarly, results are not determined by histologic types of breast tissue (medullary, invasive ductal, fibroadenoma, normal tissue). A study of fibroadenoma tissue from immunosuppressed and non-immunosuppressed patients was positive for EBV using PCR but none of the fibroadenomas from non-immunosuppressed patients expressed LMP-1 protein, suggesting that EBV may be associated with fibroadenomas only in immunocompromised patients[41]. It has been suggested that EBV detected in breast tissue samples is present in lymphocytes rather than tumour cells[28, 52, 54] but others dispute this [14, 16, 38]. Finding EBV in infiltrating lymphocytes could be expected if breast cancer is, even in part, a response to an abnormal immune stimulus.

It has been suggested that geographic differences may partly explain varying findings for EBV; nasopharyngeal carcinomas (NPC) are usually associated with EBV in populations in Hong Kong but a lower proportion of EBV-associated NPC is found in European and central Chinese populations[53]. However, no such pattern appears evident in studies of EBV, CMV, and breast cancer. Age-standardised incidence rates of breast cancer are highest in Western Europe, the USA, and Australasia, and lowest in East Asia and the Middle East.[1, 66] Most of the studies of EBV and CMV were carried out in high-risk countries but there was no clear difference between high- and low-risk countries, with the majority of studies reporting positive PCR results. The 10 studies reporting null results included high-risk areas such as the USA, Europe, and Australia but also lower-risk countries such as Iran and Mexico. Two studies compared results from different countries: one, comparing results for Egyptian and Iraqi women, found 45% of 40 Egyptian samples and 28% of 50 Iraqi samples positive.[48]; the other included 509 breast cancer samples from countries with varying risks of NPC (which is known to be associated with EBV), but found no difference in EBV positivity by geography.[16]

Another possibility is that co-infection with multiple viruses increases the risk of breast cancer. EBV may be oncogenic in conjunction with other viruses:[49] sequences from more than one virus (EBV, HPV, and MMTV) were detected in 72% of breast tumour tissue and in 13% of breast-milk samples from women without breast cancer. Tsai et al[11] using PCR to detect EBV, CMV, human papilloma virus (HPV), herpes simplex virus (HSV-1 and HSV-2), and human herpes virus-8 (HHV-8), found that, among viral-gene-positive breast cancer samples, 23% were positive for one virus, 31% were positive for two, 40% were positive for three, and 6% were positive for four viruses. In a reanalysis of these data, [67] DNA belonging to two or more viruses was more common in breast cancer and fibroadenoma (77% and 100%, respectively) than DNA from one virus (23% and 0%, respectively), suggesting that multiple viral infections may be associated with benign or malignant breast tumours. In contrast, Antonsson et al[34] investigated the prevalence of ten polyomaviruses and two herpes viruses in breast cancer using QPCR. They failed to detect eight of the 12 viruses (including CMV) in 54 breast cancer samples tested, with the highest prevalence being EBV (detected in 10% of the samples).

A recent review of the molecular evidence for viruses and human breast cancer examined the evidence for EBV.[64] This review identified three possible problems with PCR analyses: false-negative results; false-positive results; and choice of specimens for comparison.[64] False-negative results may occur because: studies that do not report DNA-quality testing cannot demonstrate that null results were not due to nucleic acid degradation; laser microdissection can cause virus levels to be too low to amplify; partial deletion or polymorphism of the viral gene could hamper hybridization; and loss of the virus during cell division may result in absence of viral DNA (‘hit and run’ behavior—discussed below). False-positive results could be caused by the presence of EBV-positive lymphocytes in tumour tissue and use of nested PCR, which is prone to contamination (although this can also occur with QPCR). The use of paired tumour and normal specimens was questioned because of the possibility that ‘normal’ samples may contain atypical cells.

Joshi and Buehring suggested that PCR studies of EBV and breast cancer that targeted BamH1W found a higher frequency of virus-presence than those targeting EBV-encoded small RNAs (EBERs),[64] but our review of the literature, which included more recent studies, does not support this. It has been demonstrated that expression of EBERs in EBV-infected cells in NPC is not universal, which may explain a low prevalence of positive results for EBERs and breast cancer, whereas EBNA-1 is always expressed in EBV infections.[68]

It is also possible that EBV and/or CMV may be associated with breast cancer through mechanisms that differ from those identified for other virus-associated cancers, including the ability of the virus to evade immunosurveillance by expressing only a few factors[63] and the possibility of a ‘hit and run’ mechanism where viral episome might be lost from malignant cells.[44, 46] Similarly, several possible mechanisms for CMV tumorigenesis have been identified, including the actions of virus-encoded interleukins, activation of telomerase, immunosuppression, and persistent infection leading to inflammation and the promotion of malignancy.[9, 25, 69] Among the studies reviewed, it was rare for EBV or CMV to be detected in 100% of specimens. It has been suggested for EBV that the ‘hit and run’ hypothesis might explain this, or that infection with EBV at a late state of tumour development might “enhance oncogenic properties, such as invasiveness, angiogenesis, and metastasis.”[46] Another plausible explanation is that either EBV or CMV is associated with a specific molecularly defined subset of breast cancers, a relationship already established for other environmental and host risk factors.[70]

## Conclusions

The results of our QPCR study support a possible association between EBV and breast cancer but not CMV and breast cancer whereas our earlier studies of CMV and EBV IgG levels found an association between elevated IgG levels and breast cancer for CMV but not EBV.[5, 6] These findings, together with the results of other studies of EBV and CMV and breast cancer, suggest several possibilities: [1] limitations of molecular analyses mean that these analyses cannot confirm whether, one, both, or neither virus is associated with breast cancer; however, sensitivity may be increased by using ISH in addition to PCR; [2] ‘hit and run’ oncogenesis means that the virus may be absent after the tumour has developed, leading to inconsistent findings; [3] one or both viruses may have a role at a later stage in the development of breast cancer—this may explain elevated CMV IgG levels associated with breast cancer; [4] infection with multiple viruses may increase the risk of breast cancer; and [5] neither virus has a role in breast cancer development. Future studies should focus on ways to investigate these possibilities and should include comparisons of breast cancer tissue samples with appropriate normal tissue samples.

## Supporting Information

S1 Table PCR Summary Table. (PDF)
