# Supplementary material for: Cytomegalovirus and Epstein-Barr Virus in Breast Cancer
Source: PLoS One. 2015 Feb 27;10(2):e0118989. doi: 10.1371/journal.pone.0118989 (PMC4344231; doi:10.1371/journal.pone.0118989)
Supplement: S1 Table — (PDF) [file pone.0118989.s001.pdf]

Table S1. PCR Summary Table

**EBV and Breast Cancer**			
**Reference**<br>**Viruses**<br>**Specimen type**<br>**Country**	**PCR type**<br>**CMV and EBV Primer(s)**<br>**Other analysis**	**Results**	**Percent EBV positive**
[1]<br>EBV<br><br>Formalin-fixed, paraffin-embedded medullary BC specimens (n=52).<br><br>USA	PCR (EBNA1)<br>Primers SL1 and SL3	35/52 specimens were suitable for PCR, but all were negative for EBV.	0 (PCR)
[2]<br>EBV<br><br>Formalin-fixed, paraffin-embedded breast carcinoma (n=3) and gastric carcinoma (n=3) tissue with severe small lymphoid cell infiltration.<br><br>Japan	PCR<br>EBV (long internal repeat 1)<br>E1 5’>CCAGACAGCAGCCAATTGTC>3’<br>E2 5’>GGTAGAAGACCCCCTCTTAC>3’<br><br>In situ hybridization (ISH)	The EBV genome was present in 2 of 3 breast cancer specimens and in 2 of 3 gastric cancer specimens.<br><br>ISH provided positive signals for the EBV genome in small lymphoid cells in 2 of 3 breast cancer specimens (the same two as above) and in 1 of 3 gastric cancer specimens (the specimen that was negative on PCR above).	66 (PCR)<br><br><br>0 (ISH)
[3]<br>EBV<br><br>Paired blood and primary breast cancer tissue samples	PCR (chose not to use nested PCR to avoid risk of contamination)<br>EBV (*Bam*HIW)<br>5'-CCATGT AAGCT-7JGCCTCG AG-3 '<br>5'-GCCTT AGATCTGGCTCTTTG-3'<br>EBV (*Bam*HIC)<br>5'-AACCTCAGGACCTACGCT-3'<br>5'-TAGCGGACAAGCCGAATAC-3’	19/91 samples (21%) were positive for the presence of the *Bam*HIW repetitive sequence. Of the 19 positive samples, 18 were available for *Bam*HIC analysis, and 15 were positive. Control samples were all negative but when 59 cycles were carried out, 3 samples became positive.	21 (PCR alone)

United Kingdom	ISH	ISH was carried out to assess whether the results obtained by PCR could be confirmed and to localise the signal. 12 of the 19 breast samples found to be positive for *Bam*HIW DNA by PCR were confirmed as positive by ISH.	63 (PCR and ISH)
[4]<br>EBV<br><br>Formalin-fixed medullary carcinoma specimens (n=10). Positive controls.<br><br>Belgium	PCR (BAM HI W)<br>5'-GCCAGTAGCATCTGACTTTGAGCCT3'<br>5'-CCACTGGCCACAAAATCA T ACAGTG 3'<br><br>IHC (LMP)<br><br>ISH	PCR: 0/10 medullary BC specimens were positive, whereas the PCR positive control was strongly positive.<br><br>IHC: 0/10 medullary BC specimens were positive (but positive controls were positive).<br><br>ISH: 0/10 medullary BC specimens were positive (but positive controls were positive).	0 (PCR)<br><br>0 (IHC)<br><br>0 (ISH)
[5]<br>EBV<br><br>Formalin-fixed, paraffin-embedded specimens; breast cancer (n=31), normal breast (n=12), Hodgkin's disease (n=1), gastric cancer (n=1), nasopharyngeal carcinoma (n=1). Breast biopsy liquid nitrogen frozen samples (n=48).<br><br>United Kingdom	PCR<br>5'GCTAGGCCACCTTCTCAG3'<br>5'GTCCAGGGCCTTCACTTC3'<br>5'CCAGCGCGTTTACGTAAG3'<br>5'TCTCCCCTAGGCTTGGAT3'<br><br>ISH (EBER)<br><br>IHC	PCR: 12 of 28 assessable breast cancers (43%) were positive. 19/48 (40%) of frozen samples were positive. 0/12 normal breast samples were positive. All the Hodgkin's disease, gastric cancer, and nasopharyngeal carcinoma specimens were positive.<br><br>ISH: None of the breast sections showed any signal. Hodgkin's disease showed a specific reaction to Reed Sternberg cells.<br><br>IHC: immunostaining was performed on a sample of the PCR-positive BC sections. The antibody produced staining of scattered tumour epithelial cells in several of the sections, which were shown to be EBV positive by PCR.	43 (PCR)<br><br>0 (ISH)
[6]<br>EBV<br><br>Samples stored in liquid nitrogen. Primary invasive breast carcinoma (n=100), normal tissues adjacent to breast tumors (n=30), and lymph nodes with metastasis (n=5).	PCR<br>GAPDH (5' to 3')<br>GGCCTCCAAGGAGTAAGACC<br>CCCCTCTTCAAGGGGTCTAC<br>BZLF1(5' to 3')<br>AGGGGAGATGTTAGACAGGT<br>AGTATGCCAGGAGTAGAACA<br>GCCACCTTTGCTATCTTTGC<br>AGGCGTGGTTTCAATAACGG	PCR: 51/100 (51%) biopsy specimens had EBV genome present. 4/5 lymph nodes with metastases had EBV genome present (for which the corresponding tumours were also EBV positive). The tumour associated with the negative lymph node was also EBV negative.<br><br>3/30 samples of healthy tissue adjacent to BC were positive for EBV (in 2 of the samples the detected genome was also observed in the tumour).	51 (PCR)

France	BNLF1(5’ to 3’)<br>CTAGCGACTCTGCTGGAAAT<br>GAGTGTGTGCCAGTTAAGGT<br>EBER-2 (5’ to 3’)<br>CCCTAGTGGTTTCGGACACA<br>ACTTGCAAATGCTCTAGGCG<br><br>Southern Blot hybridization (SBH)<br><br>ISH (EBER)<br><br>IHC (EBNA-1)	Seven BC samples were tested using SBH, and all were positive for EBV. The signal observed was independent of the lymphoplasmocytic reaction of the tumour.<br><br>ISH was performed on three EBV PCR-positive tumours and on one lymph node with metastasis. One NPC biopsy specimen served as a positive control. No signal was observed in breast tissue.<br><br>IHC was performed on six EBV-negative and on nine EBV-positive tumors with two different EBNA-1 MAbs, 1H4 and 2B4. The proportion of EBNA-1-positive cells varied greatly from one tumor to another. No labelling was seen in lymphocytes, even in lymph nodes with metastases.	0 (ISH)
[7]<br>EBV<br><br>Snap-frozen BC biopsy samples (n=24).<br><br>Netherlands	PCR (*Bam*HI-W)<br><br>Reverse transcription PCR (EBNA1 and rightward transcripts from the *Bam*HI-A region)	PCR: 5/24 (21%) positive. Of these five, two also tested positive for the single-copy gene BNLF1. In none of the five DNA PCR-positive breast carcinoma samples could EBNA1 transcripts or rightward transcripts from the *Bam*HI-A region be detected.	21 (PCR)<br><br>0 (reverse transcription PCR)
[8]<br>EBV<br><br>Paired fresh frozen and paraffin-embedded breast cancer specimens (n=48).	PCR<br>(EBNA-4)<br>5’-GAG GAG GAA GAC AAG AGT GG-3’<br>5’-GAT TCA GGC GTC GTG GCT CTT GG-3’<br>(EBV-LMP-1)<br>5’-CGG AAG TTG AAA ACA AA-3’<br>5’-GTG GGG GTC GTC ATC ATC TC-3’	PCR: 5/48 (10%) of BC specimens were positive for EBV-LMP-1, of which 3 co-expressed EBV-LMP-1 and EBNA-4. An additional 2 cases expressed either EBV-LMP-1 or EBNA-4.	10 (PCR)

USA	ISH (EBER-1)<br><br>IHC (EBNA-1, LMP-1, and ZEBRA)<br><br>Southern Blot	ISH: 5/48 (10%) of BC specimens showed nuclear and cytoplasmic positivity for EBER-1. The positive cells were tumour cells.<br><br>IHC: 12/48 (25%) of BC specimens were positive for EBNA-1. EBNA-1 did not stain any lymphocytes, endothelial cells, or stromal cells. 0/48 specimens were positive for LMP-1 or ZEBRA proteins.<br><br>Southern Blot: None of the 7 PCR positive specimens was positive.	10 (ISH)<br><br>10 (IHC)
[9]<br>EBV<br><br>Breast tumour specimens stored in liquid nitrogen (frozen and paraffin-embedded sections used for ISH). Primary invasive ductal breast carcinomas from NPC high-risk, intermediate-risk, and low-risk areas (n=509).<br><br>Algeria, Tunisia, Southern France, Northern France, Netherlands, Denmark	Qualitative *Bam*HIC PCR<br>5′-AACCTCAGGACCTACGCT-3′<br>5′-TAGCGGACAAGCCGAATAC-3′<br>Semi-quantitative *Bam*HIC PCR<br>Real-time *Bam*HIC PCR<br>5′-AAA-CAGGAC-AGC-CGT-TGC-C-3′<br>5′-AAGCCT-CTC-TTC-TCC-TTC-CCC-3′<br><br>ISH<br>Laser capture microdissection (LCM)	Overall, 31.8% of the 509 tumour samples were positive for the *Bam*HIC non repetitive sequence. Frequencies were not different among the geographical areas studied, but EBV-positive breast cancers from the NPC high- and intermediate-risk areas showed higher loads of the EBV genome than those from the low-risk areas (p = 0.01).<br><br>ISH to determine whether EBV expression occurs in the tumour epithelial compartment or in lymphoplasmocytic cells infiltrating the stroma showed positive results on the malignant epithelial compartment of tumours with more than 1500 *Bam*HIC copies/500 ng DNA (n=10). LCM confirmed the epithelial location of of EBV.	32 (PCR)<br><br>50 (ISH on 10 samples)
[10]<br>EBV<br><br>BC specimens with microdissectable components (n=115). Intraductal carcinoma (n=84), invasive tumor (n=106), metastases (n=50), recurrences, normal lymph node, or other normal tissue (total n=361).	PCR (EBNA1)<br>CTGGAAATGGCCTAGGAGAGAA (nucleotides 637–658;GenBank S45894)<br>TCCATGGTTATCACCCCCTC (nucleotides 709–728)<br><br>ISH (EBER, LMP1)	Amplifiable DNA was obtained from 278/361 (77%) samples. Of these, only six samples were positive for EBNA1.<br><br>EBV-positive blocks were analysed by EBER ISH and LMP1 immunohistochemistry. Three were lymph nodes, and all of these contained occasional EBER- and LMP1-positive lymphocytes; the metastatic tumor itself was	2 (PCR)

USA		negative for both EBER and LMP1.<br><br>The other 3 positive specimens were from one patient. Both EBER and LMP1 staining demonstrated EBV was present in 50%–75% of normal ductal epithelium, as well as in the intraductal and invasive carcinoma components.	
[11]<br>EBV<br><br>Formalin-fixed, paraffin-embedded; infiltrating ductal (n=20), infiltrating lobular (n=13), fibroadenoma (n=10), fibrocystic changes (n=16).<br><br>USA	PCR (EBER)<br>5' to 3'<br>CCCTAGTGGTTTCGGACACA<br>ACTTGCAAATGCTCTAGGCG<br><br>ISH (EBER)	In 14/33 (42%) carcinomas, convincing nuclear reactions were noted. 21 normal breast, non-proliferative variants of fibrocystic changes, and benign fibroadenomas were negative. CD21 showed no reaction in any epithelial component.	42 (IHC)<br><br>Of these, 100% were positive on PCR
[12]<br>EBV<br><br>Formalin-fixed, paraffin-embedded, invasive carcinoma; 30 invasive ductal, 26 invasive lobular, 2 medullary, 1 undifferentiated (total n=59) Control; paraffin sections from EBV-positive undifferentiated nasopharyngeal carcinoma.<br><br>Germany	PCR<br>EBV *Bam*HI W<br>5'-CAC TTT AGA GCT CTG GAGGA-3'<br>5'-TAA AGA TAG CAG CAG CAC AG-3'<br><br>ISH<br><br>IHC (EBNA-1)	After 35 amplification cycles, a PCR product of the expected size was observed in four of 59 cases (6.8%). Subsequent analysis of the EBV DNA PCR-positive cases by EBV DNA<br><br>ISH using a 35S-labelled probe specific for the EBV *Bam*HI W fragment yielded negative results, while the same approach led to a strong labelling of nasopharyngeal carcinoma cell nuclei.	7 (PCR)<br><br><br><br><br>0 (ISH)
[13]<br>EBV<br><br>Formalin-fixed, paraffin-embedded tissue samples from rapidly-growing breast fibroadenomas in immunosuppressed (n=20) & non-	PCR (EBER-2)<br>5'-CCCTAGTGGTTTCGGACACA-3'<br>5'-ACTTGCAAATGCTCTAGGCG-3'	EBV genome was detected through PCR amplification of EBER-2 DNA, in 13 of 18 (72%) fibroadenomas, and LMP-1 protein in 12 of 20 (60%). None had lymphocytic infiltration, which minimizes the possibility that the positive EBER-2 results were due to contaminating lymphocytes.	72 (PCR)

immunosuppressed<br>(n=11) patients.<br><br>USA	Real-time PCR (nested primers)<br>5’-ATCTTCGGGTGCTTACTTG-3’<br>5’-AAGGCCAAAAGCTGCCAGATGGTGGC-3’<br>5’-CATTGTTCCTTGGAATTGTGCTGT-TC-3’<br>5’-ACCAAGTCGCCAGAGCATC-3’<br><br>Immunohistochemistry (IHC)	LMP-1 RNA was not detected using nested RT-PCR.<br><br>Nine (45%) were concordantly positive for both, EBER-2 DNA and LMP-1 protein. None of the fibroadenomas from non-immunosuppressed patients expressed LMP-1 protein (*P* =0.0006).	45 (PCR and IHC)
[14]<br>EBV<br><br>Paraffin-embedded samples of breast cancer NST (n=84), DCIS (n=8), medullary carcinoma (n=4), atypical medullary carcinoma (n=1), and mucinous type (n=1).<br><br>United Kingdom	Real-time PCR<br>EBV (polymerase gene)<br>5’-AGTCCTTCTTGGCTAGTCTGTTGAC-3’<br>5’-CTTTGGCGCGGATCCTC-3’<br>EBV (β-*2m* gene)<br>5’-GGAATTGATTTGGGAGAGCATC-3’<br>5’-CAGGTCCTGGCTCTACAATTTACTAA-3’<br><br>LCM<br><br>ISH (EBER)<br><br>IHC using 2B4-1 reagent)	Six tumour samples excluded. EBV DNA was detected in 19 of 92 cases (21%), but in all cases, the genome copy number was very low (mean of 1.1 copies of EBV/1000 cells; range, 0.1–7.1). LCM was used to isolate tumour cells from the 19 positive samples. Subsequent Q-PCR of these samples failed to detect EBV DNA in any of these (although positive controls were positive).<br><br>EBER ISH for EBERS did not detect RNAs in tumour cells from any of the 19 samples (but strong reactivity was seen in positive controls).<br><br>Positive results were found for 2B4-1 reagent in 19/25 (76%) samples negative for EBV genome. The 2B4-1 protein is similar to EBNA-1.	21 (QPCR)<br><br>0 (PCR after LCM)<br><br>0 (ISH)
[15]<br>EBV<br><br>Breast epithelial tumour cell lines<br><br>USA	RT-PCR<br>EBV (EBER1, BARF0, RPMS1, EBNA1, Wp/Cp, LMP1, ZTA) Table 1 lists primers.	In vitro-infected breast carcinoma cells expressed a mixture of latency II and lytic EBV genes. Both BZLF1 transcripts and Zta protein expression were detected, and 10% of the EBV converted MDA-MB468-BX1 cells exhibited spontaneous Zta expression in immunofluorescence assays.	70 (QPCR)
[16]<br>EBV	PCR<br>EBER-1 and EBER-2<br>50-AGGACCTACGCTGCCCTAGA	6/15 (40%) of the BC samples were positive for EBV early gene LF3 (which contains the repetitive IR4 sequence).	40 (PCR)

Fresh frozen biopsies Ductal carcinoma (n=12), anaplastic lobular (n=1), lobular with ductal elements (n=1), tubule-lobular (n=1).<br><br>United Kingdom	and 50-AAAACATGCGGACCACCAGC,<br>50-CCCTAGTGGTTTCGGACACAC<br>and 50-GACAAGCCGAATACCCTTCTC<br>CST/BART<br>50-TGCGCCTGGAAGTTGTACTCCCGGAA<br>and 50-CTACCGCCACGCGTCAGCAAA<br><br>IHC	6/6 (100%) of EBV-positive breast cancers were positive for EBV CST/BART and LF3/IR4 transcripts.	
[17]<br>EBV<br><br>Formalin-fixed, paraffin-embedded breast cancer; DCIS (n=28), invasive lobular (n=20), various malignant (n=9). Controls: normal breast tissue/benign lesions (n=55).<br><br>Turkey	PCR<br>EBV (GP220 gene)<br>5' -GGC TGG TGT CAC ATC TGT TA-3'<br>5' -CCT TAG GAG GAA CAA GTC CC-3'	Of the 57 breast carcinoma and 55 benign/normal samples, 13 (23%) and 19 (35%) were positive for EBV DNA, respectively. Of the 28 invasive ductal, 20 invasive lobular and 9 various malignant lesions, 7 (25%), 4 (30%), and 2 (22%) were positive for EBV DNA, respectively. As few as 0.1 copies of EBV per cell in 0.1 mg of DNA were detected.	23 (PCR)
[18]<br>EBV<br><br>Matched normal and breast carcinoma (infiltrating ductal n=37, infiltrating lobular n=4, tubular carcinoma n=3, mucinous carcinoma n=1) formalin-fixed, paraffin-embedded samples from 45 patients.<br><br>USA	QPCR<br>5V-TCACCCACACTGTGCCCATCTACGA-3V<br>5V-TGAGGTAGTCAGTCAGGTCCCG-3V,probe<br>5VATGCCCTCCCCCATGCCATCCTGCGT-3V;<br>forward primer<br>5V-TGACCTACTTGGACCATGTGGA-3V,<br>reverse primer<br>5V-TGATGAGACTTCCGAGTGCACT-3V, probe<br>5VCAGTGTCCTGATCCTGGACCTTGACTATG<br>AA-3V; BALF 5,<br>forward primer 5V-<br>CGGAAGCCCTCTGGACTTC-3V, reverse primer<br>5V-CCCTGTTTATCCGATGGAATG-3V, probe<br>5VTGTACACGCACGAGAAATGCGCC-<br>3V.<br><br>ISH (EBER)	In no case could EBV DNA be consistently detected, with either of 2 different probes, at levels above 0.1 molecules per cell. The distribution of these low signals was the same between tumour tissues and the matched normal controls.	0 (QPCR)

[19]<br>EBV<br><br>Breast cancer biopsy specimens (n=69). Controls: normal breast tissue/benign lesions (n=48).<br><br>Argentina	PCR<br>EBV (*Bam*HIW)<br>CCAGAGGTAAGTGGACTT<br>GACCGGTGCCTTCTTAGG<br><br>EBNA-1 immunostaining	12/39 fresh tissue samples (31%) were positive on PCR.<br><br>24/69 samples (35%) were positive for EBNA-1. None of the control samples was positive.	31 (PCR)<br><br>35 (IHC)
[20]<br>EBV<br><br>Formalin-fixed, paraffin-embedded invasive ductal carcinoma samples (n=55).<br><br>USA	Real-time PCR<br>EBV (BamH1W, LMPI, EBNA1, LMP2, BZLF1)<br><br>ISH<br>IHC<br>LCM	Low level EBV DNA (up to 11 copies per 100,000 cells) was detected in 4/55 cancers (7%) by at least one Q-PCR assay.<br><br>LCM used to separate neoplastic from non-neoplastic cells in the 4 positive specimens found no EBV DNA in either (while control assay showed adequate human DNA amplification). IHC and ISH of the 4 specimens were also negative (but positive for positive controls).	7 (QPCR)<br><br>0 (IHC after LCM))<br><br>0 (ISH after LCM)
[21]<br>EBV<br><br>Frozen adenocarcinoma specimens (n=95)<br><br>France	Q-PCR<br>EBNA-1 E1AS TTGCAGCCAATGCAACTTGG<br>E1S AGAGAGTAGTCTCAGGGCAT<br>BamH1U UPUS GTTCCTCGGTGGCGGGCTTA<br>ExonUAS U172AS<br>ATGCCCTGAGACTACTCTCT<br>LMP-1 LMP1S CTGAGGATGGAACACGACCT<br>LMP1AS AATGGAGGGAGAGTCAGTCA<br>BARF-1 BARF1S<br>GGCTGTCACCGCTTTCTTGG<br>BARF1AS AGGTGTTGGCACTTCTGTGG<br>BamH1A BamAS<br>CGTGGTGAAGCCTCTAACGC<br>BamAAS GGCAAGTGCGTTTATTGCGA<br>BZLF1 ZS TTACACCTGACCCATACCAG<br>ZAS1 ACATCTGCTTCAACAGGAGG<br>HPRT HPRTS TATGGACAGGACTGAACGTC<br>HPRTAS GTTGAGAGATCATCTCCACC<br>Southern Blot hybridization<br>LCM	Q-PCR assays were performed on 95 whole samples of breast cancer biopsy tissues. In 51/95 (54%) no copies of the EBV genome were amplified or the number was below the threshold of detection. 44/95 (46%) samples were positive for EBV.	46 (QPCR)

[22]<br>EBV<br><br>Fresh frozen invasive breast carcinoma samples and blood samples from 24 patients. Breast cancer cell lines (n=14)<br><br>USA	Real time PCR<br>EBV (*Bam* HIW)<br>5'-CCC AAC ACT CCA CCA CAC C-3'<br>5'-TCT TAG GAG CTG TCC GAG GG-3'	11/24 (46%) were positive for EBV DNA. Of these 11, 7 (64%) were also EBV positive in the peripheral blood, and the other 4 negative positive tumor samples were negative in the paired blood specimens. Conversely, 3 of 10 (30%) patients were positive for EBV DNA in the peripheral blood but this was undetected in the tumour. Overall, 10 of the 24 (42%) patients' peripheral blood specimens were positive for EBV DNA while 11 (46%) were positive for EBV in the tumour DNA.<br>EBV viral DNA was undetectable in the BC cell lines.	46 (QPCR)
[23]<br>EBV<br><br>Sections from paraffin blocks; primary unilateral invasive breast cancer (n=40) and controls with fibrocystic disease (n=20)<br><br>Egypt	PCR<br>EBV (EBNA-1)<br>5′-GTTCGCGTTGCTAGGCCACC-3′<br>5′-AGGACCACTTTATACCAGGG-3′<br><br>IHC	8/40 (20%) BC samples were positive on PCR, and 32/40 (80%) were negative.<br>0/20 controls were positive on PCR and 20/20 (100%) were negative.<br><br>10/40 (25%) BC samples were positive on IHC and 30/40 (75%) were negative<br>0/20 controls were positive on PCR and 20/20 (100%) were negative.	20 (PCR)
[24]<br>EBV<br><br>Frozen breast cancer and adjacent normal tissues (n=123)<br><br>Tunisia	PCR<br>EBV (*Bam*HIG)<br>3'-AACATGCTGTATGCCTCGCAGCG-5'<br>3'-AATTACTGGCGTGAATTGTGCCCA-5'<br><br>ISH (EBER)<br>IHC	EBV DNA was found in 27% (33/123) of breast carcinoma cases. None of the normal breast tissues showed the presence of EBV DNA.<br><br>ISH was negative in neoplastic cells, but some stromal infiltrating lymphocytes were positive in tumor tissues of 4 cases. IHC for LMP1 was negative in all tumor tissues. Neither ISH nor IHC showed any positive EBV results in normal breast tissues.	27 (PCR)<br><br>0 (ISH)<br><br>0 (IHC)
[25]<br>EBV<br><br>Formalin-fixed, paraffin-embedded invasive breast carcinoma samples (n=100). Controls were benign and normal breast tissue (n=42)	PCR<br>EBV (EBER)<br>CCCTAGTGGTTTCGGACACACA<br>ACTTGCAAATGCTCTAGGCG<br>EBV (*Bam*HIW)<br>CCAGAGGTAAGTGGACTT<br>GACCGGTGCCTTCTTAGG	Amplification fragments of either 108 bp from the EBER regions or 122 bp from the *Bam*HIW region of EBV were not detected by PCR in any of the breast carcinoma or benign breast lesions.<br><br>None of the breast carcinoma or benign lesions specimens which were subjected to IHC showed nuclear staining	0 (PCR)

Iran	IHC (EBNA-2 and LMP-1)	which is typical for EBNA2 and none of them showed membrane staining which is typical for LMP1 expression.	
[26]<br>EBV<br><br>Frozen primary invasive breast cancer and lymph node tissue (n=196)<br><br>France	Q-PCR<br>EBV (*Bam*HIC)<br>5'-AAA-CAG-GAC-AGC-CGT-TGC-C-3'<br>5'-AAG-CCT-CTC-TTC-TCC-TTC-CCC-3'	EBV was detected in 65 (33.2%) of the 196 investigated BCs. Among the positive tumours, the load of EBV genome varied from 0.08 to 810.8 *Bam*HIC copies per 100 ng GAPDH (median 1.4).<br><br>Normal tissue and phyllode tumour tissues were all found to be negative, with the exception of one phyllode tumour (0.43 *Bam*HIC copies per 100 ng GAPDH).	33 (QPCR)
[27]<br>EBV<br><br>Paraffin-embedded primary invasive breast cancer from Egyptian (n=40) and Iraqi (n=50) women<br><br>Egypt, Iraq	PCR<br>EBV (EBNA1)<br>EBV (LMP1)<br>(Primers not stated)<br>ISH (EBER)<br>IHC	PCR-Egyptian positive cases were 23/40(57.5%). Of those 23 patients, 18(45%) were positive for EBER(ISH). From those 18 patients, 12(30%) cases were positive for LMP1 (PCR), suggesting latent EBV infection.<br><br>PCR-Iraqi positive cases were 16/50(32%). Of those 16 patients, 14(28%) were positive for EBER (ISH), and 11(22%) cases were positive for LMP1 (PCR), suggesting latent infection.	58 (PCR Egyptian samples)<br><br>32 (PCR Iraqi samples)
[28]<br>EBV, HPV, MMTV<br><br>Fresh frozen DNA extracts from unselected breast cancers (n=50) epithelial cells in human milk from lactating women without BC (n=40) Archival formalin-fixed breast cancer (n=27) and normal breast (n=18) tissue<br><br>Australia	Standard PCR<br>EBV(EBNA-1 gene)<br>Two sets of primers:<br>5'-AAGGAGGGTGGTTTGGAAAG<br>5'-AGACAATGGACTCCCTTAGC<br><br>5'-ATCGTGGTCAAGGAGGTTCC<br>5'-ACTCAATGGTGTAAGACGAC<br><br>In situ PCR<br><br>IHC (LMP1 and EBNA-1)	EBV was detected in 34 (68%), HPV (all HPV 18 by sequencing) in 25 (50%) and MMTV in 39 (78%) of the 50 fresh frozen, invasive breast cancer specimens tested by standard PCR. EBV was detected in 14 (35%), HPV (all HPV 18 by sequencing) in 8 (20%) and MMTV in 13 (32%) of epithelial cells from 40 samples of milk from normal lactating women.<br><br>Multiple viruses in the same breast cancer specimen were identified frequently.	68 (PCR)
[29]<br>EBV	In situ PCR (*Bam* H1W)<br>50–30 (bp 14,224–14,245)<br>TGTGACTTCACCAAAGGTCAGG	EBV detected in *leukocytes* of 13/70 (18.6%) of tissue samples using ISH and in 1/70 (1.4%) using IS-PCR.	

Formalin-fixed, paraffin-embedded breast cancer tissues (n=70)<br><br>USA	30–50 (bp 14,298–14,280)<br>ACGTAAACGCGCTGGACTG<br><br>ISH (EBER)	EBV not detected in mammary epithelium of any specimens by ISH and in only 2/70 (2.9%) using IS-PCR. Both of these specimens were low grade ductal carcinomas and the positive reaction was in areas of normal lobules.	0 (ISH)<br><br>3 (IS-PCR)
[30]<br>EBV, MMTV<br><br>Frozen BC (n = 86) and adjacent tissue samples (n=65) from Mexico City, Formalin-fixed, paraffin-embedded BC and adjacent tissue samples from Yucatan (n=21)<br><br>Mexico	Nested PCR<br>EBV (*Bam*HIW)<br>5' CTT TGT CCA GATGTC AGG GG 3'<br>5' GCC TGA GCC TCT ACT TTT GG 3'	There were no positive samples after the first PCR, but 4/86 (4.7%) positive samples with the nested PCR. None of the adjacent non-tumor tissues were EBV positive after the first and nested PCRs.<br>(Nested PCR was designed to test a number of tumor cells exceeding the sensitivity of the first PCR).	0 (PCR)<br>5 (nested PCR)
**CMV (or CMV and EBV) and Breast Cancer**			
**Reference**<br>**Viruses**<br>**Specimen type**<br>**Country**	**PCR type**<br>**CMV and EBV Primer(s)**<br>**Other analysis**	**Results**	**Percent CMV and/or EBV positive**
[31]<br>CMV, EBV, HPV, HSV-1, HSV-2, HHV-8<br><br>Fresh invasive ductal breast cancer, breast fibroadenoma, benign thyroid tumour, and normal tissue stored in liquid nitrogen until used for this study.<br><br>Taiwan	PCR<br>CMV (IE2 region)<br>IE2-1: 5'-TCCTCCTGCAGTTCGGCTTC-3'<br>IE2-2: 5'-TTTCATGATATTGCGCACCT-3'<br>EBV (*Bam*HI-W fragment)<br>a: 5'-TCGCGTTGCTAGGCCACCTT-3'<br>b: 5'-CTTGGATGGCGGAGTCAGCG-3'<br><br>Each sample tested by PCR three times. The DNA was confirmed by Southern hybridization.	Of 62 breast cancer samples, 8 (12.9%) were positive for HSV-1, 28 (45.2%) for EBV, 47 (75.8%) for CMV, 8 (12.9%) for HPV, and 28 (43.8%) for HHV-8<br><br>The presence of multiple viruses was common in the breast cancer and fibroadenoma groups, but was not detected in the non-cancerous and thyroid tumour groups. Only CMV was detected in some of the tissues of non-cancerous or thyroid tumours.	76 CMV (PCR)<br><br>45 EBV (PCR)

[32]<br>CMV, EBV, HPV, HSV-1, HSV-2, HHV-8<br><br>Same 62 breast cancer samples as above.	As above.	Relapse-free survival was calculated using the period from the first day of surgery until the day of relapse or last follow-up visit. Overall survival was calculated from the first day of surgery until death or last follow-up visit. HSV-1, HHV-8, EBV, CMV, and HPV were related to overall survival, but only HHV-8 and CMV were related to relapse-free survival ($P < 0.05$ or $P < 0.01$)	CMV and EBV related to overall survival.<br><br>CMV related to relapse-free survival.
[33]<br>CMV<br><br>Formalin-fixed, paraffin-embedded surgical biopsy specimens of 21 paired breast cancer and normal tissue (from breast cancer patients) and 38 normal tissue specimens (from reduction mammoplasty patients).<br><br>USA	100-250 ng of DNA was amplified by nested PCR using internal and external primers specific for HCMV glycoprotein B (UL55) gene. Confirmation of HCMV sequence was performed using a NCBI Blast search.<br><br>Immunohistochemistry (IHC)<br><br>In-situ hybridization (ISH)	Nested PCR testing of 8 tumours (all of which were positive for CMV by IHC) and 4 control samples (1 of which was positive for CMV by IHC) found that 6/8 tumour cases and 1/4 normal control cases demonstrated amplified CMV UL55 gene, which was confirmed by direct sequencing of the PCR products.<br><br>IHC detected CMV-IE antigen expression in glandular epithelium in 63% of normal breast samples, but in 97% of ductal carcinoma in situ and infiltrating ductal carcinoma samples (p = 0.0009).<br><br>ISH detected CMV nucleic acids in 16/18 randomly selected breast cancer specimens, 3/3 randomly selected 'control' specimens, and 11/18 randomly selected 'normal control' specimens.	75 (nested PCR)<br><br>97 (IHC)<br><br>89 (ISH)
[34]<br>CMV, EBV, SV40, BKV, JCV, MCV, WUV, KIV, LPV, HPyV6, HPyV7, TSV<br><br>54 fresh frozen breast cancer specimens, and 10 paired adjacent tumour free breast tissue specimens<br><br>Australia	Real-time PCR<br>CMV (MIE protein)<br>AAC TCA GCC TTC CCT AAG ACC A<br>CAA TGG CTG CAG TCA GGC CAT GG<br>EBV (BALF5 gene)<br>5'-CGGAAGCCCTCTGGACTTC-3'<br>5'-CCCTGTTTATCCGATGGAATG-3'	Tested for 12 DNA viruses. The highest prevalence, 10% (5/54), was found for EBV. MCV, HPyV6, and HPyV7 were detected in single patient samples (2% each), while WUV, KIV, JCV, BKV, LPV, SV40, TSV and CMV were not detected in the 54 specimens.	0 (QPCR)
[35]<br>CMV<br><br>Paraffin-embedded breast	PCR<br>5'-GTC ACC AAG GCC ACG ACG TT-3' 5'-TCT GCC AGG ACA TCT TTC TC-3'	2 of 24 (8.3%) breast carcinoma samples and 0 of 24 fibroadenoma samples were positive on PCR.	8 (PCR)

carcinoma (n=24) and fibroadenoma (n=24) samples<br><br>Iran			
[36]<br>CMV<br><br>Formalin-fixed, paraffin-embedded specimens from breast cancer and paired sentinel lymph nodes (SLN) in patients with (n=35) and without SLN metastases (n=38).<br><br>Sweden	TaqMan real-time PCR<br>CMV (IE-gene)<br>GTGACCCATGTGCTTATGACTCTAT<br>CTCAACATAGTCTGCAGGAACGT<br><br>IHC	12 breast cancer and 16 SLN specimens tested using PCR. CMV DNA was detected in 12/12 (100%) of breast cancer specimens and in 10/11 (91%) of metastatic SLN specimens. CMV DNA was not detected in any of the 5 SLN-negative specimens.<br><br>CMV proteins were expressed in 100% of primary breast cancer specimens, in 32/34 (94%) SLN-positive specimens, and in 20/35 (60%) of SLN-negative specimens. CMV protein expression was mainly confined to metastatic tumour cells, but also in inflammatory cells in SLN samples.	100 (QPCR)<br><br>100 (IHC)
[37]<br>CMV<br><br>Fresh tissue samples from patients with inflammatory BC (n=28) and non-inflammatory BC (n=49)<br><br>Egypt	Nested PCR<br>CMV (Fourth exon of IE gene)<br>External primers<br>5'GGTCACTAGTGACGCTTGTATGATGA-3'<br>5'-GATAGTCGCGGGTACAGGGGACTCT-3'; Internal primers<br>5'-AAGTGAGTTCTGTCGGGTGCT-3'<br>5'-GTGACACCAGAGAATCAGAGGA-3'	CMV is associated with inflammatory breast cancer. 53.1% of non-IBC carcinoma tissues were HCMV-DNA positive while in IBC patients 78.6% of carcinoma tissues were HCMV-DNA positive (p= 0.030).<br><br>In control samples (number not stated) 16.7% of non-carcinoma tissues of non-IBC patients were HCMV-DNA positive. All of the examined non-carcinoma tissues of IBC patients (100%) and healthy breast tissues (100%) were HCMV DNA negative.	Non-IBC 53 (nested PCR)<br><br>IBC 79 (nested PCR)
[38]<br>CMV<br><br>Paraffin-embedded breast tissue; stages II, III, and IV (n=27) and fibroadenoma tissue (n=20)<br><br>Mexico	Real-time PCR<br>CMV (*Ie2*, UL122)<br>GGCTCACCTCGTCAATCTTGA<br>AGAAGGTGCGCAATATCATGAAAGA<br>CMV (*pp65*, UL83)<br>GGGACACAACACCGTAAAGC<br>GTGGAAGAGGACCTAACGATGAC	2/27 tumours (7.4%) were positive for CMV, with all the remaining samples negative. One sample was positive for both *Ie2* 'early gene' (synonym of viral replication) and *pp65* 'late gene' (presence of virus), while the other was positive for the late gene only.	7 (QPCR)

Richardson et al 2014<br>CMV, EBV<br><br>Paired breast cancer and normal tissue samples (n=70)<br><br>New Zealand	Real-time PCR<br>CMV pp-65<br>GCAGCCACGGGATCGTACT<br>GGCTTTTACCTCACACGAGCATT<br>EBNA-1 (primers unavailable)<br><br>Serology	EBV was detected in 24/70 tumour specimens (34%) and in 9/70 paired normal specimens (13%).<br><br>CMV was not detected in any tumour specimens. CMV was detected in 2/70 paired normal specimens (3%).<br><br>72/76 (95%) serology samples were EBV IgG positive.<br>52/76 serology samples (68%) were CMV IgG positive.	34 EBV<br>(QPCR)<br><br>0 CMV<br>(QPCR)

References:

1. Gaffey MJ, Frierson HF, Jr., Mills SE, Boyd JC, Zarbo RJ, et al. (1993) Medullary carcinoma of the breast. Identification of lymphocyte subpopulations and their significance. Mod Pathol 6: 721-728.
2. Horiuchi K, Mishima K, Ohsawa M, Aozasa K (1994) Carcinoma of stomach and breast with lymphoid stroma: localisation of Epstein-Barr virus. J Clin Pathol 47: 538-540.
3. Labrecque LG, Barnes DM, Fentiman IS, Griffin BE (1995) Epstein-Barr virus in epithelial cell tumors: a breast cancer study. Cancer Res 55: 39-45.
4. Lespagnard L, Cochaux P, Larsimont D, Degeyter M, Velu T, et al. (1995) Absence of Epstein-Barr virus in medullary carcinoma of the breast as demonstrated by immunophenotyping, in situ hybridization and polymerase chain reaction. Am J Clin Pathol 103: 449-452.
5. Luqmani Y, Shousha S (1995) Presence of epstein-barr-virus in breast-carcinoma. Int J Oncol 6: 899-903.
6. Bonnet M, Guinebretiere JM, Kremmer E, al e (1999) Detection of Epstein-Barr virus in invasive breast cancers. Journal of the National Cancer Institute 91: 1376-1381.
7. Brink AA, van Den Brule AJ, van Diest P, Meijer CJ (2000) Re: detection of Epstein-Barr virus in invasive breast cancers. J Natl Cancer Inst 92: 655-656; author reply 656.
8. Chu PG, Chang KL, Chen YY, Chen WG, Weiss LM (2001) No significant association of Epstein-Barr virus infection with invasive breast carcinoma. Am J Pathol 159: 571-578.
9. Fina F, Romain S, Ouafik L, Palmari J, Ben Ayed F, et al. (2001) Frequency and genome load of Epstein-Barr virus in 509 breast cancers from different geographical areas. Br J Cancer 84: 783-790.
10. McCall SA, Lichy JH, Bijwaard KE, Aguilera NS, Chu WS, et al. (2001) Epstein-Barr virus detection in ductal carcinoma of the breast. J Natl Cancer Inst 93: 148-150.
11. Grinstein S, Preciado MV, Gattuso P, Chabay PA, Warren WH, et al. (2002) Demonstration of Epstein-Barr virus in carcinomas of various sites. Cancer Res 62: 4876-4878.
12. Herrmann K, Niedobitek G (2003) Lack of evidence for an association of Epstein-Barr virus infection with breast carcinoma. Breast Cancer Res 5: R13-17.
13. Kleer CG, Tseng MD, Gutsch DE, Rochford RA, Wu Z, et al. (2002) Detection of Epstein-Barr virus in rapidly growing fibroadenomas of the breast in immunosuppressed hosts. Mod Pathol 15: 759-764.
14. Murray PG, Lissauer D, Junying J, Davies G, Moore S, et al. (2003) Reactivity with A monoclonal antibody to Epstein-Barr virus (EBV) nuclear antigen 1 defines a subset of aggressive breast cancers in the absence of the EBV genome. Cancer Res 63: 2338-2343.
15. Huang J, Chen H, Hutt-Fletcher L, Ambinder RF, Hayward SD (2003) Lytic viral replication as a contributor to the detection of Epstein-Barr virus in breast cancer. J Virol 77: 13267-13274.

16. Xue SA, Lampert IA, Haldane JS, Bridger JE, Griffin BE (2003) Epstein-Barr virus gene expression in human breast cancer: protagonist or passenger? Br J Cancer 89: 113-119.
17. Kalkan A, Ozdarendeli A, Bulut Y, Yekeler H, Cobanoglu B, et al. (2005) Investigation of Epstein-Barr virus DNA in formalin-fixed and paraffin- embedded breast cancer tissues. Med Princ Pract 14: 268-271.
18. Perrigoue JG, den Boon JA, Friedl A, Newton MA, Ahlquist P, et al. (2005) Lack of association between EBV and breast carcinoma. Cancer Epidemiol Biomarkers Prev 14: 809-814.
19. Preciado MV, Chabay PA, De Matteo EN, Gonzalez P, Grinstein S, et al. (2005) Epstein-Barr virus in breast carcinoma in Argentina. Arch Pathol Lab Med 129: 377-381.
20. Thorne LB, Ryan JL, Elmore SH, Glaser SL, Gulley ML (2005) Real-time PCR measures Epstein-Barr Virus DNA in archival breast adenocarcinomas. Diagn Mol Pathol 14: 29-33.
21. Arbach H, Viglasky V, Lefeu F, Guinebretiere JM, Ramirez V, et al. (2006) Epstein-Barr virus (EBV) genome and expression in breast cancer tissue: effect of EBV infection of breast cancer cells on resistance to paclitaxel (Taxol). J Virol 80: 845-853.
22. Perkins RS, Sahm K, Marando C, Dickson-Witmer D, Pahnke GR, et al. (2006) Analysis of Epstein-Barr virus reservoirs in paired blood and breast cancer primary biopsy specimens by real time PCR. Breast Cancer Res 8: R70.
23. Fawzy S, Sallam M, Awad NM (2008) Detection of Epstein-Barr virus in breast carcinoma in Egyptian women. Clin Biochem 41: 486-492.
24. Hachana M, Amara K, Ziadi S, Romdhane E, Gacem RB, et al. (2011) Investigation of Epstein-Barr virus in breast carcinomas in Tunisia. Pathol Res Pract 207: 695-700.
25. Kadivar M, Monabati A, Joulaee A, Hosseini N (2011) Epstein-Barr virus and breast cancer: lack of evidence for an association in Iranian women. Pathol Oncol Res 17: 489-492.
26. Mazouni C, Fina F, Romain S, Ouafik L, Bonnier P, et al. (2011) Epstein-Barr virus as a marker of biological aggressiveness in breast cancer. Br J Cancer 104: 332-337.
27. Zekri AR, Bahnassy AA, Mohamed WS, El-Kassem FA, El-Khalidi SJ, et al. (2012) Epstein-Barr virus and breast cancer: epidemiological and molecular study on Egyptian and Iraqi women. J Egypt Natl Canc Inst 24: 123-131.
28. Glenn WK, Heng B, Delprado W, Iacopetta B, Whitaker NJ, et al. (2012) Epstein-Barr virus, human papillomavirus and mouse mammary tumour virus as multiple viruses in breast cancer. PLoS One 7: e48788.
29. Baltzell K, Buehring GC, Krishnamurthy S, Kuerer H, Shen HM, et al. (2012) Epstein-Barr virus is seldom found in mammary epithelium of breast cancer tissue using in situ molecular methods. Breast Cancer Res Treat 132: 267-274.
30. Morales-Sanchez A, Molina-Munoz T, Martinez-Lopez JL, Hernandez-Sancen P, Mantilla A, et al. (2013) No association between Epstein-Barr Virus and Mouse Mammary Tumor Virus with breast cancer in Mexican women. Sci Rep 3: 2970.
31. Tsai JH, Tsai CH, Cheng MH, Lin SJ, Xu FL, et al. (2005) Association of viral factors with non-familial breast cancer in Taiwan by comparison with non-cancerous, fibroadenoma, and thyroid tumor tissues. J Med Virol 75: 276-281.

32. Tsai J, Hsu C, Tsai C, al e (2007) Relationship between viral factors, axillary lymph node status and survival in breast cancer. J Cancer Res Clin Ocol 133: 13-21.
33. Harkins LE, Matalaf LA, Soroceanu L, al e (2010) Detection of human cytomegalovirus in normal and neoplastic breast epithelium. Herpesviridae 1: 8.
34. Antonsson A, Bialasiewicz S, Rockett RJ, Jacob K, Bennett IC, et al. (2012) Exploring the prevalence of ten polyomaviruses and two herpes viruses in breast cancer. PLoS ONE 7.
35. Eghbali M, Ghane M, Mirinargesi M (2012) Frequency of cytomegalovirus (CMV) in benign and malignant tumors. International Journal of Molecular and Clinical Microbiology 2: 175-179.
36. Taher C, de Boniface J, Mohammad AA, Religa P, Hartman J, et al. (2013) High prevalence of human cytomegalovirus proteins and nucleic acids in primary breast cancer and metastatic sentinel lymph nodes. PLoS One 8: e56795.
37. El-Shinawi M, Mohamed HT, El-Ghonaimy EA, Tantawy M, Younis A, et al. (2013) Human cytomegalovirus infection enhances NF-kappaB/p65 signaling in inflammatory breast cancer patients. PLoS One 8: e55755.
38. Utrera-Barillas D, Valdez-Salazar HA, Gomez-Rangel D, Alvarado-Cabrero I, Aguilera P, et al. (2013) Is human cytomegalovirus associated with breast cancer progression? Infectious Agents & Cancer [Electronic Resource] 8: 12.
